# The relationship of soluble TREM2 to other biomarkers of sporadic Alzheimer’s disease

**DOI:** 10.1038/s41598-021-92101-6

**Published:** 2021-06-22

**Authors:** So-Hee Park, Eun-Hye Lee, Hyung-Ji Kim, Sungyang Jo, Sunju Lee, Sang Won Seo, Hyun-Hee Park, Seong-Ho Koh, Jae-Hong Lee

**Affiliations:** 1 Seongnam Center of Senior Health, Seongnam-si, Gyeongi-do, 13200 Republic of Korea; 2 Department of Neurology, Hanyang University Guri Hospital, Hanyang University College of Medicine, Gyeongchun-ro, Guri-Si, Gyeonggi-do, 11923 Republic of Korea; 3 Department of Neurology, University of Ulsan College of Medicine, Asan Medical Center, 88 Olympic-ro 43-gil, Songpa-gu, Seoul, 05505 Republic of Korea; 4 Department of Neurology, Samsung Medical Center, Sungkyunkwan University School of Medicine, Seoul, 06351 Republic of Korea

**Keywords:** Neuroscience, Biomarkers, Neurology

## Abstract

Microglial activation is a central player in the pathophysiology of Alzheimer’s disease (AD). The soluble fragment of triggering receptor expressed on myeloid cells 2 (sTREM2) can serve as a marker for microglial activation and has been shown to be overexpressed in AD. However, the relationship of sTREM2 with other AD biomarkers has not been extensively studied. We investigated the relationship between cerebrospinal fluid (CSF) sTREM2 and other AD biomarkers and examined the correlation of plasma sTREM2 with CSF sTREM2 in a cohort of individuals with AD and without AD. Participants were consecutively recruited from Asan Medical Center from 2018 to 2020. Subjects were stratified by their amyloid positivity and clinical status. Along with other AD biomarkers, sTREM2 level was measured in the plasma as well as CSF. In 101 patients with either amyloid-positive or negative status, CSF sTREM2 was closely associated with CSF T-tau and P-tau and not with Abeta42. CSF sTREM2 levels were found to be strongly correlated with CSF neurofilament light chain. The comparison of CSF and plasma sTREM2 levels tended to have an inverse correlation. Plasma sTREM2 and P-tau levels were oppositely influenced by age. Our results suggest that neuroinflammation may be closely associated with tau-induced neurodegeneration.

## Introduction

Biomarkers have now become an essential component of Alzheimer’s research. Extracellular amyloid plaque and intraneuronal hyperphosphorylated tau accumulation are considered hallmarks of Alzheimer’s disease (AD) and many biomarkers have been developed to reflect these central pathophysiological events. By virtue of these biomarkers, we possess a valuable window into the changes occurring in the brain of individuals with Alzheimer’s disease and can identify the disease process at the earliest possible stage^1^.

Neuroinflammation has been deemed a secondary phenomenon, but is now emerging as a central player in the development of AD^2–4^. This theory is centered on microglial activation, which can be brought about by various stimuli, including beta-amyloid. Failure of activated microglia to wall off amyloid plaques from surrounding neurons and phagocytize them leads to the accumulation of amyloid plaques and neurofibrillary tangles^5,6^. Microglial activation is mediated by its membrane-bound receptor, triggering receptor expressed on myeloid cells 2 (TREM2)^7^. The activity of TREM2 can be measured by evaluating the levels of the soluble fragment of TREM2 (sTREM2), which is generated during the TREM2 cleavage process^8^. The concentration of sTREM2 in the cerebrospinal fluid (CSF) has been shown to be elevated in AD, raising the possibility that sTREM2 can serve as a reliable biomarker for AD^9–11^. It is important to investigate at the relationship of one biomarker with another to better understand the pathophysiology of AD. The role of microglial activation in the amyloid cascade is a matter of curiosity and requires further exploration^12^. However, the relationship between sTREM2 and other conventional biomarkers across the various stages of AD has not been extensively studied, particularly in patients with sporadic AD. Whether the change in CSF sTREM2 can be reflected in the plasma is also controversial^13,14^. If sTREM2-induced changes in the brain can be reflected in the plasma levels of sTREM2 akin to neurofilament light chain in multiple sclerosis, we will be able to elucidate neuroinflammation associated with AD throughout the course of the disease.

The purpose of this study was to investigate the relationship between CSF sTREM2 and other AD biomarkers and to examine the plasma levels of sTREM2 and its correlation with CSF sTREM2 in a cohort population of AD and non-AD conditions.

## Results

### Demographics

The clinical characteristics of the participants are presented in Table 1. The amyloid-positive and amyloid-negative groups showed a significant difference in age (P = 0.025). The mean age of the amyloid-positive group, in which 21 early-onset patients are included, was lower than that of the amyloid-negative group (66.7 vs. 71.2 year). The frequency of the *APOE* ε4 allele was significantly higher in the amyloid-positive group than that in the amyloid-negative group (P = 0.003, hazard ratio = 16.57). The frequency of diabetes mellitus was higher in the amyloid-negative group than that in the amyloid-positive group (P = 0.01). The amyloid-positive group had poor performances in K-MMSE compared to those of the amyloid-negative group (P = 0.029). The amyloid-positive group showed significantly higher serum LDL levels (P = 0.042) and albumin (P = 0.02). The amyloid-negative group showed higher serum ESR than did the amyloid-positive group (P = 0.025). Other demographic features, including sex distribution, CRP serum levels, frequency of hypertension (HTN), and hyperlipidemia, did not show significant differences between the two groups.

**Table 1:** Demographics and clinical characteristics of amyloid-negative and amyloid-positive groups.

	Amyloid-negative (N = 47)	Amyloid-positive (N = 54)
**Population**		
Sex, n (female)	47 (22)	54 (32)
Age (SD), year*	71.18 (8.66)	66.73 (10.36)
Education level (SD), year	11.76 (4.67)	11.41 (4.77)
Disease duration (SD), months	28.84 (28.12)	35.62 (27.10)
*APOE ε4* carrier, n (%)**	9 (18.8)	30 (57.7)
**Underlying disease**		
DM, n (%)*	22 (44.9)	10 (19.2)
HTN, n (%)	22 (44.9)	25 (48.1)
Hyperlipidemia, n (%)	20 (40.8)	26 (50.0)
**Global cognition**		
MMSE (SD)*	24.12 (4.65)	22.00 (4.90)
CDR (SD)	0.67 (0.52)	0.72 (0.45)
**Laboratory test**		
Neutrophil count, % (SD)	56.46 (8.81)	56.64 (8.91)
Monocyte count, % (SD)	8.13 (1.61)	11.05 (19.01)
ESR (SD), mm/h*	15.47 (11.41)	10.91 (7.00)
CRP (SD)	0.23 (0.45)	0.21 (0.48)
LDL (SD), mg/dL*	104.78 (38.70)	127.03 (38.21)
Albumin (SD), g/dL*	3.69 (0.39)	3.84 (0.26)

Demographics and characteristics of the dataset.

Student’s *t*-test was used for analyzing the age, educational level, disease duration, MMSE, CDR, neutrophil, monocyte, ESR, CRP, and LDL levels.

χ^2^ test was used in the analysis of sex distribution, *APOE ε4*, DM, HTN, and hyperlipidemia.

*DM* diabetes mellitus, *HTN* hypertension, *ESR* erythrocyte sedimentation rate, *CRP* C-reactive proteins, *LDL* low-density lipoprotein.

*Significant at P < 0.05.

**Significant at P < 0.01.

### AD biomarkers in the cohort

**Figure 1:**
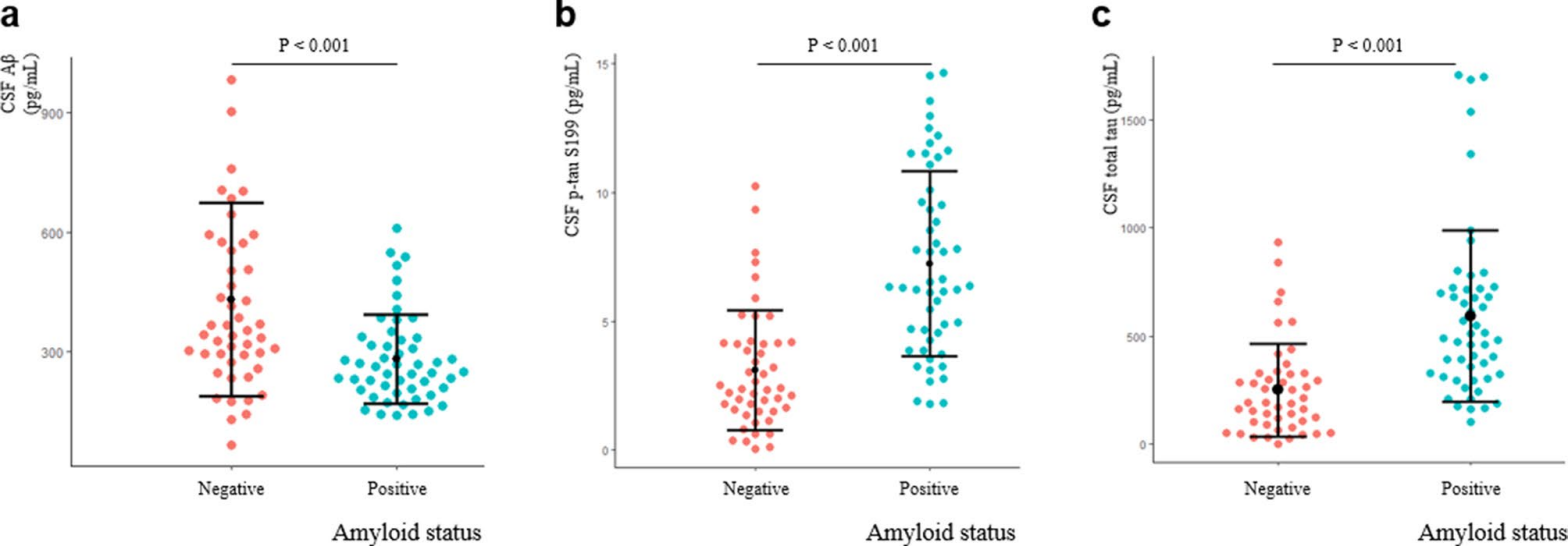
Alzheimer’s disease (AD) biomarkers in the cohort. To demonstrate that the cohort had typical AD biomarker patterns, the levels of CSF Aβ_42_, T-tau, and P-tau S199 were compared between the two groups. Aβ_42_ levels were significantly lower in the amyloid-positive group than those in the amyloid-negative group (**a**). The cerebrospinal fluid (CSF) P-tau S199 (**b**) and T-tau (**c**) levels were significantly higher in the amyloid-positive group than those in the amyloid-negative group. Statistical analysis was performed using the Mann–Whitney test. *sTREM2* soluble triggering-receptor expressed on myeloid cells 2, *NfL* neurofilament light chain, *NRGN* neurogranin. Statistical significance *P < 0.05, **P < 0.01.

The CSF Aβ_42_, T-tau, and P-Tau S199 levels were compared between the two groups (amyloid-positive and amyloid-negative) to demonstrate that the cohort had typical patterns of AD core biomarkers. As expected, the CSF Aβ_42_ levels were significantly lower in the amyloid-positive group than those in the amyloid-negative group (P < 0.001) (Fig. 1a). The CSF P-Tau S199 (P < 0.001) and T-tau (P < 0.001) levels were significantly higher in the amyloid-positive group than those in the amyloid-negative group (Fig. 1b,c).

### CSF and plasma sTREM2 levels, CSF and plasma biomarkers

**Figure 2:**
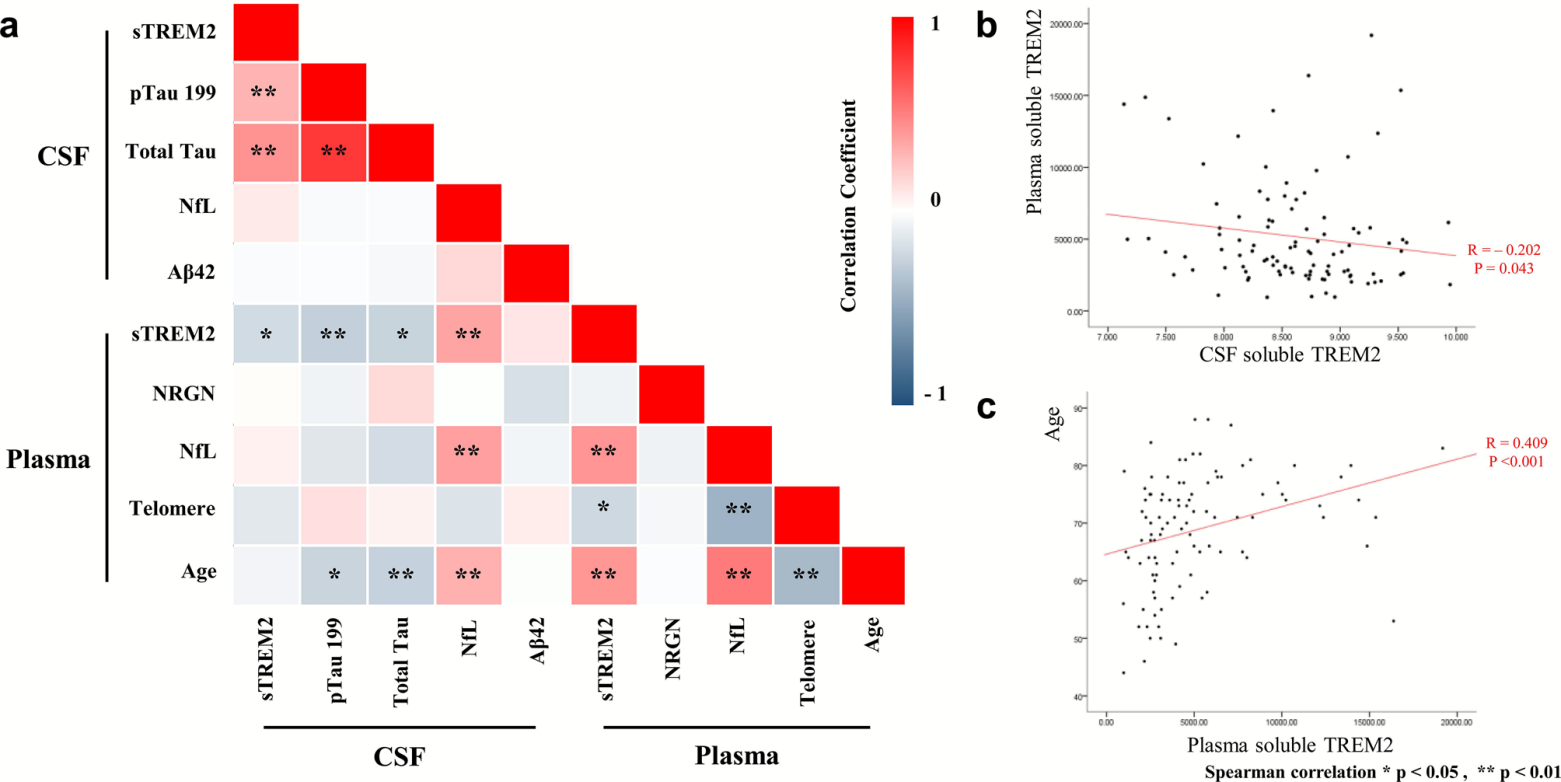
Relationship among biomarkers as a whole dataset. The relationship between cerebrospinal fluid (CSF) and plasma biomarkers is displayed in the heatmap (**a**). The levels of soluble triggering-receptor expressed on myeloid cells 2 (sTREM2) in CSF and plasma were negatively correlated (**b**). Age was also positively correlated with the plasma sTREM2 levels (**c**). Statistical analysis was performed by linear regression analysis. *sTREM2* soluble triggering-receptor expressed on myeloid cells 2, *NfL* neurofilament light chain, *NRGN* neurogranin. Statistical significance *P < 0.05, **P < 0.01.

In the whole group, the CSF sTREM2 levels were negatively correlated with the plasma sTREM2 levels (Spearman's rho [ρ] = – 0.202, P = 0.043; n = 101) (Fig. 2a,b). The CSF sTREM2 levels positively correlated with the CSF P-Tau S199 (ρ = 0.296, P = 0.003; n = 100) and T-tau levels (ρ = 0.427, P < 0.001; n = 99) (Fig. 2a). In contrast, the plasma sTREM2 levels were negatively correlated with the CSF P-tau S199 (ρ = – 0.264, P = 0.008; n = 100) and T-tau levels (ρ = – 0.248, P = 0.013; n = 99)  (Fig. 2a). Plasma sTREM2 levels were significantly correlated with the CSF NfL levels (ρ = 0.359, P < 0.001; n = 101) (Fig. 2a). The CSF sTREM2 levels were not significantly correlated with the CSF NfL levels (ρ = 0.085, P = 0.401; n = 101). With respect to the age effects, age correlated positively with the plasma sTREM2 levels (ρ = 0.409, P < 0.001; n = 101) and negatively with the CSF P-tau S199 (ρ = – 0.240, P = 0.016; n = 100) and T-tau levels (ρ = – 0.258, P = 0.010; n = 99) (Fig. 2c).

The plasma sTREM2 levels were positively correlated with the plasma NfL levels (ρ = 0.416, P < 0.001; n = 100). In contrast, the CSF sTREM2 was not significantly correlated with the plasma biomarkers (Table 2 and Fig. 2a).

**Table 2:** Correlation of cerebrospinal fluid (CSF) and plasma soluble triggering receptor expressed on myeloid cells 2 (sTREM2) with Alzheimer’s disease (AD) biomarkers in all subjects (N=101).

		**CSF sTREM2**		**Plasma sTREM2**	
		R	P-value	R	P-value
CSF	Aβ42	– 0.021	0.835	0.104	0.303
	P-tau S199	0.296	0.003	-0.264	0.008
	T-tau	0.427	< 0.001	-0.248	0.013
	Aβ42/P-tau S199	– 0.273	0.006	0.267	0.007
	NfL	0.085	0.401	0.359	< 0.001
Plasma	NfL	0.056	0.582	0.416	< 0.001
	NRGN	0.012	0.906	– 0.068	0.512
	Telomere	– 0.127	0.205	– 0.216	0.030
	Age	– 0.057	0.573	0.409	< 0.001

Linear regression analyses were used to demonstrate a correlation between biomarkers.

*NfL* neurofilament light chain, *NRGN* neurogranin.

### Plasma neurodegeneration biomarkers

The plasma sTREM2 positively correlated with plasma NfL (ρ = 0.416, P < 0.001; n = 100) and age (ρ = 0.409, P < 0.001; n = 101). Furthermore, NfL and age were strongly correlated with each other (ρ = 0.523, P < 0.001; n = 100). In contrast, the leukocyte TL was negatively correlated with sTREM2 (ρ = – 0.216, P < 0.03; n = 101), plasma NfL (ρ = – 0.429, P < 0.001; n = 100), and age (ρ = – 0.387, P < 0.001; n = 101), showing that the aging and neurodegeneration factors were progressing together (Fig. 2).

### CSF and plasma sTREM2 in the AD continuum

**Figure 3:**
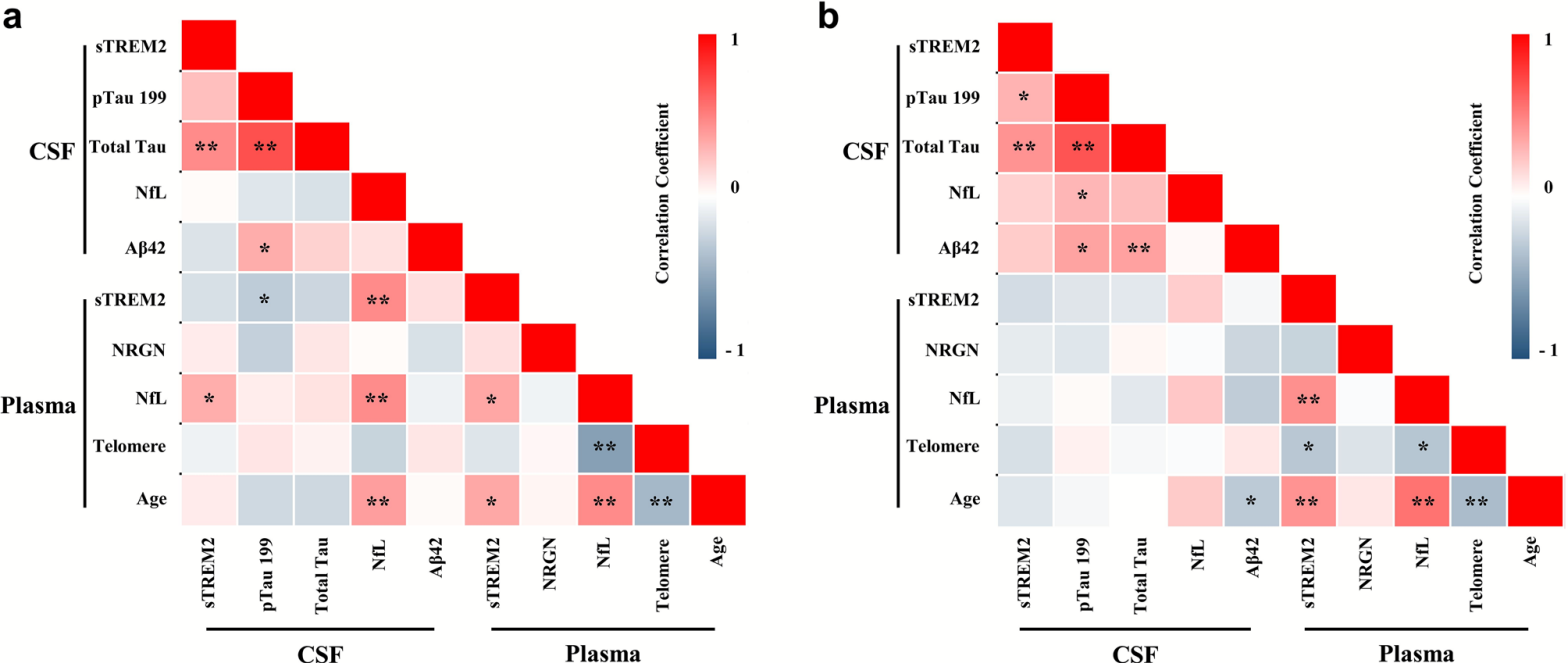
Relationship between biomarkers in the amyloid-positive and -negative groups. The relationship between cerebrospinal fluid (CSF) and plasma biomarker in the amyloid-positive group is displayed in the heatmap (**a**). The relationship between CSF and plasma biomarker in the amyloid-negative group is displayed in the heatmap (**b**). Statistical analysis was performed by linear regression analysis. *sTREM2* soluble triggering-receptor expressed on myeloid cells 2, *NfL* neurofilament light chain, *NRGN* neurogranin. Statistical significance *P < 0.05, **P < 0.01.

In the amyloid-positive group (AD continuum), the association between CSF and plasma sTREM2 which was observed in the whole dataset disappeared. The CSF sTREM2 levels significantly correlated with CSF T-tau level (ρ = 0.455, P = 0.0014; n = 51), and Aβ42/P-tau S199 (ρ = – 0.331, P = 0.017; n = 52). The plasma sTREM2 levels correlated with CSF P-tau S199 (ρ = –  0.288, P = 0.038; n = 52) and Aβ_42_/P-tau S199 (ρ = 0.316, P = 0.022; n = 52), CSF NfL (ρ = 0.457, P = 0.001; n = 52), and plasma NfL (ρ = 0.349, P = 0.012; n = 51). The plasma sTREM2 levels correlated positively with age (ρ = 0.345, P = 0.012; n = 52) (Table 3 and Fig. 3a).

**Table 3:** Correlation of cerebrospinal fluid (CSF) and plasma soluble triggering receptor expressed on myeloid cells 2 (sTREM2) with Alzheimer’s disease (AD) biomarkers in patients with AD continuum (amyloid-positive group) (N=47).

		**CSF sTREM2**		**Plasma sTREM2**	
		R	P-value	R	P-value
CSF	Aβ42	– 0.152	0.281	0.128	0.365
	P-tau S199	0.254	0.069	-0.288	0.038
	T-tau	0.455	< 0.001	-0.227	0.110
	Aβ42/P-tau S199	– 0.331	0.017	0.316	0.022
	NfL	0.018	0.901	0.457	<0.001
Plasma	NfL	0.323	0.021	0.349	0.012
	NRGN	0.080	0.584	0.126	0.389
	Telomere	– 0.072	0.610	– 0.146	0.303
	Age	0.080	0.574	0.345	0.012

Linear regression analyses were used to demonstrate a correlation between biomarkers.

*NfL* neurofilament light chain, *NRGN* neurogranin.

### CSF and plasma sTREM2 in non-AD condition

In the amyloid-negative conditions, the CSF sTREM2 levels showed a positive correlation with the CSF P-tau S199 (ρ = 0.301, P = 0.038; n = 48) and T-tau (ρ = 0.425, P = 0.003; n = 48) levels. No significant significance was found between the plasma sTREM2 and CSF biomarker levels. The plasma sTREM2 levels were correlated with the plasma NfL (ρ = 0.436, P = 0.002; n = 49), TL (ρ = – 0.312, P = 0.029; n = 49), and age (ρ = 0.430, P = 0.002; n = 49) (Table 4 and Fig. 3b).

**Table 4:** Correlation of cerebrospinal fluid (CSF) and plasma soluble triggering receptor expressed on myeloid cells 2 (sTREM2) with Alzheimer’s disease (AD) biomarkers in the amyloid-negative group (N=54).

		**CSF sTREM2**		**Plasma sTREM2**	
		R	P-value	R	P-value
CSF	Aβ42	0.204	0.159	–0.041	0.779
	P-tau S199	0.301	0.038	–0.142	0.335
	T-tau	0.425	0.003	–0.126	0.394
	Aβ42/P-tau S199	– 0.178	0.225	0.115	0.438
	NfL	0.183	0.208	0.196	0.177
Plasma	NfL	–0.083	0.572	0.436	0.002
	NRGN	–0.109	0.465	– 0.246	0.095
	Telomere	–0.175	0.230	– 0.312	0.029
	Age	–0.140	0.338	0.430	0.002

Linear regression analyses were used to demonstrate a correlation between biomarkers.

*NfL* neurofilament light chain, *NRGN* neurogranin.

### Relationship of CSF P-tau and T-tau with age stratified by amyloid status

**Figure 4:**
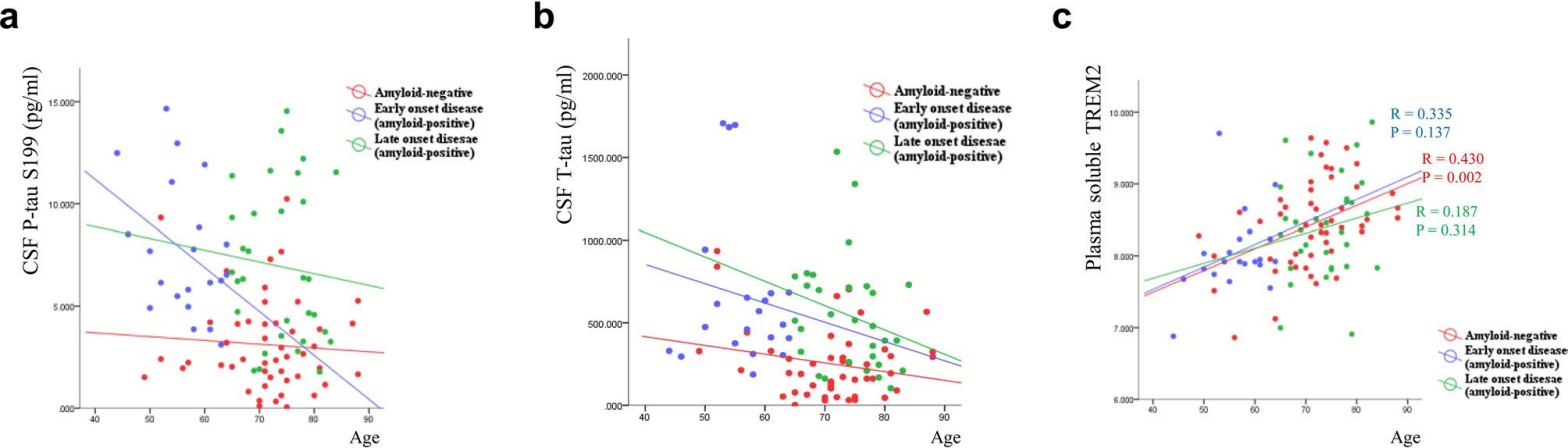
Effect of age on biomarkers. All participants were categorized into three groups: amyloid-negative, amyloid-positive early-onset (younger than age 65), and amyloid-positive late-onset. The tau pathology over the age course was negatively correlated with CSF P-tau S199 only in the early-onset amyloid-positive group, but this was not significant (**a**,**b**). In the case of plasma sTREM2, the amyloid-negative group still showed a strong positive correlation with age (**c**). Statistical analysis was performed by linear regression analysis.

To avoid age bias, all participants were categorized into three different groups, i.e., amyloid-negative, amyloid-positive early-onset (younger than age 65), and amyloid-positive late-onset disease. In case of CSF P-Tau S199, T-tau levels, and age correlation, we found that the CSF P-tau S199 and T-tau levels were inversely influenced by age in the whole dataset, as previously noted (Fig. 3). The tau pathology over the age course was negatively correlated with CSF P-tau S199 only in the early-onset amyloid-positive group, but this was not significant (Fig. 4a,b). In case of plasma sTREM2, the amyloid-negative group still showed a strong positive correlation with age (ρ = 0.430, P = 0.002). However, no significance was observed in the amyloid-positive early or late onset group (Fig. 4c).

## Discussion

We collected both plasma and CSF samples from participants in this AD biomarker study to examine whether CSF sTREM2 can be mapped onto other AD biomarkers and also correlate with plasma sTREM2. The major findings of this study were as follows: first, we observed that CSF sTREM2 was closely associated with CSF T-tau and P-tau, but not so much with Aβ_42_; second, the CSF sTREM2 levels were found to be strongly correlated with CSF NfL; third, comparison of the CSF sTREM2 and plasma sTREM2 levels tended to show an inverse correlation; fourth, the plasma sTREM2 and P-tau levels were influenced by age in an opposite way (higher levels of sTREM2 and lower levels of P-tau were associated with older age). Taken together, these results suggest that sTREM2 can serve as a reliable biomarker of AD and that neuroinflammation may be closely associated with tau-induced neurodegeneration.

With regard to the relationship of sTREM2 to well-known AD biomarkers, a longitudinal study of the DIAN cohort revealed that CSF sTREM2 levels were higher in patients with MCI due to AD than in AD and control groups^9^. Their levels were closely associated with P-tau levels but not with Aβ_42_ levels. The role of sTREM2 in AD is contentious^2,6,15–17^. It could be either a byproduct of full-length TREM2 shedding or an active peptide playing a role in microglial modulation^18,19^. The increase in CSF sTREM2 levels in the early symptomatic stage of AD was interpreted to reflect a corresponding change in status of microglial activation in response to neurodegeneration induced by hyperphosphorylated tau aggregation^8^. Another cross-sectional study of sporadic AD patients showed that the levels of sTREM2 peaked at the early symptomatic phase of the disease and that the CSF sTREM2 levels were positively correlated with the concentrations of T-tau and P-tau in the CSF, suggesting the important role of sTREM2 in the development of AD pathology and neurodegeneration^9,20,21^. We can suggest that elevated levels of sTREM2 may have close relationship to inducing tau phosphorylation in neurites neighboring amyloid plaques^22,23^. Microglial clustering around amyloid plaques represented by increased sTREM2 levels play a role in initially providing a barrier function^24^. If it fails to wall off amyloid plaques from surrounding neurons and phagocytize them, this can lead to the propagation of amyloid plaques and formation of neurofibrillary tangles^25,26^. Dystrophic neurites containing hyperphosphorylated tau can be found around amyloid plaques during this process^27^. Why CSF sTREM2was not significantly associated with Aβ_42_ in our sample is unknown. Given the early appearance of Aβ_42_ and its plateau in the prodromal stage of AD, giving way to the increase in P-tau, sTREM2 may be closely linked with Aβ-triggered tau phosphorylation occurring after Aβ_42_ accumulation^11,28^. Tau aggregations might affect the microglial activation state, suppressing Aβ clearance and producing neurodegeneration^29,30^. sTREM2 could serve as a marker for the tau-dependent pathogenetic pathway of AD^31,32^.

Plasma sTREM2 levels were found to be strongly correlated with CSF neurofilament light chain (NfL). These results are in accordance with those of prior studies^33^. Increased levels of NfL are thought to represent axonal damage induced by neuroinflammatory conditions, typically exemplified by multiple sclerosis^34^. These two proteins moving in the same direction indicate that sTREM2 can reflect microglial activation and consequent neuroinflammation. The comparison of CSF sTREM2 and plasma sTREM2 levels revealed no definite relationship with AD continuum. The CSF sTREM2 levels showed a trend toward having an inverse correlation with the plasma sTREM2 levels. This disconnect between CSF sTREM2 and plasma sTREM2 is somewhat difficult to explain. In multiple sclerosis, there is a strong positive correlation between CSF NfL and plasma NfL, indicating the same direction for peripheral and central neuroinflammation^13,35^. In AD, however, the relationship between CSF sTREM2 and plasma sTREM2 is not straightforward, and previous reports on this are inconsistent^36–38^. One study performed on AD showed a significant association between peripheral sTREM2 and CSF sTREM2 levels^13^. The opposite results were reported in a study performed on AD and healthy subjects, where increased levels of CSF sTREM2 were observed in AD patients, particularly in the early stage; however, plasma levels were not different between the two groups, suggesting incorrect plasma sTREM2 levels^11,39^. In AD, the plasma sTREM2 levels may not necessarily reflect the CSF sTREM2 levels, and may not follow the same dynamics as CSF sTREM2^33,40^. Further studies are required to clarify this.

It is well known that there is an age-related increment in tau pathology, as with the increasing production of tau protein in the brain of aging individuals^41–43^, however the different way that age impacts the CSF sTREM2 and tau levels is intriguing^44,45^. Higher levels of plasma sTREM2 were associated with older age, whereas lower levels of tau were correlated with older age. Aging per se is represented by a chronic and systemic low-grade inflammatory process. In this context, increased levels of plasma sTREM2, a potential neuroinflammatory biomarker, are not surprising in older subjects, but decreased levels of CSF P-tau and T-tau in the elderly are quite puzzling, even more so given the apparent positive correlation between the CSF sTREM2 and tau levels. These are the findings derived from the group as a whole across the amyloid and clinical status. When we focused on the AD continuum, however, the age effect on the CSF tau levels disappeared^46^. As we broke down the subjects into earlier onset (< 65 years) and late onset AD (Fig. 4), we found that this inverse relationship was primarily driven by the earlier onset AD patients. It is well known that EOAD has higher AD pathology burden including P-tau^47–51^. Since a significant proportion of earlier onset AD subjects happened to be enrolled into our study, this age disproportion was likely to have affected the results, giving rise to the spurious finding.

Our study has several limitations. First, this is a single-center prospective study; thus, sampling bias might exist. Second, with many groups present based on the amyloid status and cognitive severity, the sample size of each group is not large enough to show sufficient statistical power; the validation of our findings through a large-scale study is required. Third, the data on biomarkers in this study are from the baseline of this cohort and longitudinal changes in sTREM2 are not available. It would be more interesting if we continued to collect follow-up data on biomarkers in this cohort to see how the sTREM2 levels fluctuate over the course of AD. Fourth, we chose P-tau S199 for tau hyperphosphorylation status over P-tau181 or P-tau217 either of which is generally preferred for phosphorylated tau measurement. Prior to this study, we verified that P-tauS199 was comparable to the other epitopes through a literature search^52,53^. Fifth, we did not incorporate the apolipoprotein E genotype into the relationship between sTREM2 and other biomarkers, which needs to be further explored^54,55^.

However, despite these limitations, to the best of our knowledge, to date, this is the first attempt to explore the importance of sTREM2 in relation to other AD biomarkers using both CSF and plasma in individuals with and without AD pathology. We are planning to analyze forthcoming longitudinal data using more competitive markers such as Aβ_42_/Aβ_40_ ratio, P-tau 181 and P-tau 231 in order to solidify our data.

## Conclusion

The results of this study suggest that soluble TREM2 may serve as a potentially useful biomarker for the microglial status and consequent neuroinflammation in the AD continuum. Thus, great emphasis should be placed on sTREM2 so as to obtain a more complete picture of AD.

## Methods

### Participants

We enrolled 104 participants prospectively from the memory clinic of Asan Medical Center, Seoul, Korea from June 2018 to July 2020. All participants or their proxies signed an informed consent form. All subjects underwent brain MRI, comprehensive neuropsychological testing, fluorine-18 [^18^F]-florbetaben amyloid positron emission tomography (PET), and CSF analysis. Subjects were included based on the following criteria: (1) aged over 40 years and under 90 years; (2) no evidence of parenchymal lesions that could influence the cognitive function based on brain MRI. Three subjects were excluded from the dataset because of the withdrawal of consent and thus a total of 101 subjects were included in this study.

All PET images were obtained using Discovery 690, 710, and 690 Elite PET/computed tomography scanners (GE Healthcare; Chicago, IL, USA). Amyloid PET images were collected for 20 min, beginning 90 min after injection of 300 ± 30 MBq ^18^F-florbetaben. Two neurologists (H.J.K and J.H.L) and two nuclear medicine physicians (J.S.K. and M.O.) reviewed the PET scans according to the predefined regional cortical tracer uptake (RCTU) and brain amyloid plaque load (BAPL) scoring system. In general, four regions of interest, comprising the frontal, temporal, and parietal cortex and posterior cingulate/precuneus, were interpreted in the visual assessment of the [^18^F]-florbetaben PET scans. The RCTU scores were then condensed into a single three-grade scoring system for each PET scan (BAPL score): 1, no β-amyloid load; 2, minor β-amyloid load; 3, significant β-amyloid load. The final score was reached by consensus, with a BAPL score of 1 regarded as amyloid negative (Aβ-) and BAPL scores of 2 and 3 considered to be amyloid positive (Aβ+)^56^. All participants were classified into one of these two groups according to the PET results.

We divided subjects into amyloid positive and negative groups according to the amyloid status on PET irrespective of the cognitive status. In each group, therefore, there exists a cognitive continuum spanning from SMI to dementia.

All of the participants underwent the following blood tests: complete blood count; lipid profile; erythrocyte sedimentation rate (ESR); vitamin B_12_, folate, and homocysteine serum level test; and thyroid function test. The apolipoprotein E (*APOE*) genotype was identified after extracting genomic DNA from the venous blood. All CSF samples were subjected to complete cell count, protein, and albumin tests.

This study was approved by the Institutional Review Board of Asan Medical Center, Republic of Korea (#2018–0614). All methods were carried out in accordance with relevant guidelines and regulations.

### Reagents

Proximity ligation assay (PLA) buffer, comprising 1 mM D-biotin (Life Technologies), 0.1% purified BSA (Bovogen), 0.05% Tween-20 (Sigma), 100 nM goat IgG (Sigma), 0.1 μg/μL salmon sperm DNA (Life Technologies), and 5 mM EDTA in PBS (pH 7.4) (Sigma), was stored at – 20 ℃. Conjugate probe sequences were provided as below. Streptavidin conjugated oligonucleotide SLC1 (Streptavidin-5′-CGCATCGCCCTTGGACTACGACTGACGAAC CGCTTTGCCTGACTGATCGCTAAATCGTG-3′) and SLC2 (5′–TCGTGTCTAAAGTCC GTTACCTTGATTCCCCTAACCCTCTTGAAAAATTCGGCATCGGTGA-3′-streptavidin) were purchased from Solulink. The ligation was conducted by using polymerase chain reaction (PCR) primer 1 (reverse) 5′-GGGAATCAAGGTAACGGACTTTAG-3′, PCR primer 2 (forward) 5′-CATCGCCC TTGGACTACGA-3, and PCR primer 3 (splint) 5′-TACTTAGACACGACACGATTTAG TTT-3′.

### Solid phase proximity ligation assay (spPLA)

To detect sTREM2 in the CSF and plasma, a spPLA was conducted following the previous protocol^57^. spPLA is able to detect proteins from 1 or 10 nM to femtomolar concentrations. sTREM2 concentrations in the CSF and plasma were measured using biotinylated anti-sTREM2 polyclonal antibody and recombinant TREM2 protein (R&D systems). Briefly, 1 mg/mL Dynabeads MyOne Streptavidin T1 (Invitrogen) was incubated with 50 nM biotinylated anti-sTREM2 polyclonal antibody (R&D systems) for 1 h at room temperature (RT) under rotation to immobilize the sTREM2 antibodies to the magnetic beads. The magnetic beads were washed twice with 0.05% Tween 20 in 1X PBS (washing buffer). During the reaction, plasma samples were diluted twofold in PLA buffer, and the recombinant TREM2 protein (R&D systems) was serially spiked in PLA buffer from 1 nM to 1 fM as a standard. The diluted plasma samples and serial dilution of the standards were then mixed with the antibody-conjugated magnetic beads and incubated for 90 min under rotation. Then, the beads were washed two times with washing buffer. The PLA probes were formed by separately incubating 50 nM of the streptavidin–oligonucleotide conjugates SLC1 and SLC2 with 50 nM biotinylated anti-sTREM2 antibody for 1 h at RT. Prior to use, the SLC1- and SLC2-anti-sTREM2 antibodies were mixed at an equal ratio and incubated for 5 min at RT. The final concentration of each probe was 500 pM. Finally, the magnetic beads were mixed with 1 nM of PLA probe mix and incubated for another 90 min at RT followed by washing twice with washing buffer. Real-time PCR mix (1X PCR buffer [QIAGEN] comprising 2.5 mM MgCl_2_ [QIAGEN], 0.22 μM Sybr Green [Life Technologies], 0.1 μM of each primer [reverse primer, forward primer, and splint primer] [IDT], 80 μM ATP [ThermoFisher], 0.2 mM dNTP with U [ThermoFisher], 0.03 U/μL Taq polymerase [QIAGEN], 0.01 U/μL T4 DNA ligase [ThermoFisher], 0.02 U/μL UNG [ThermoFisher], and nuclease-free water [QIAGEN]) was prepared as previously described. Before the cycling stage, a heat incubation step was performed for 15 min at 95 °C, and application steps were performed at 40 cycles for 30 s at 94 °C, 1 min at 50 °C, and 1 min at 72 °C. A StepOnePlus real-time PCR instrument (Applied Biosystems) was used for the experiment and analysis. To investigate the performance efficiency of sTREM2 spPLA, LLOD, LLOQ, precision, and dilutional linearity were measured. The LLOD formula was CtLOD = CtN-2SN. CtN is the average of Ct value gained from the background noise and SN is the standard deviation of this value. sTREM2 spPLA had inter-assay precision < 17.5% and intra-assay precision < 2%. In terms of inter-assay precision, previous study explained that the relatively large coefficients of variation are the results of the PCR step, which can be highly variable at low target copy numbers. The sTREM2 spPLA showed LLOD 50.58 pg/mL, LLOQ 151.75 pg/mL and average 95.9% dilutional linearity.

### Plasma and CSF biomarkers

To measure the levels of the biomarkers, we conducted ELISA and SIMOA following the instructions provided from commercial suppliers. For p-tau S199 measurement, Tau (Phospho) pS199 Human ELISA Kit (Invitrogen) was performed with CSF samples. Briefly, standards and samples were spiked with standard diluent buffer at 1:1 ratio and added 96 wells plate coated with capture antibodies against P-Tau S199 for 2 h. Then, unattached antigens were washed out. The human P-Tau S199 detection antibody solution applied to the wells for 1 h following 30 min of incubation with anti-rabbit IgG HRP solution. Finally, the chromogen was added to each well and incubated for 30 min. The reaction was stopped with stop solution and read the absorbance at 450 nm. For total-tau in CSF, human tau (Total) ELISA kit (Invitrogen) was conducted. The standards and samples were diluted with standard diluent buffer at 1:1 ratio and added to the wells coated with capture antibodies for 2 h at room temperature. After washing, human total tau biotin conjugate antibodies were added to each well for 1 h. Then, streptavidin-HRP solution was applied to the wells for 30 min. Finally, stabilized chromogen and stop solutions were added to each well. The level of total tau in CSF was read at 450 nm. In case of Aβ42 levels, the CSF was diluted threefold with sample diluent due to high endogenous levels. The standards and samples were added and incubated for 2 h at 2–8 °C. After 4 times of washing, human amyloid β (aa1-42) conjugate added to each well and incubated for another 2 h at at 2–8 °C. The wells were washed out and substrate was applied and reacted with for 30 min at room temperature. The level of Aβ42 was determined using a microplate reader (Biotek) set to 450 nm.

For plasma neurogranin, Human Neurogranin ELISA (Lifespan Biosciences) was used. Samples were applied to the wells for 1 h. After washing step, detection reagent A and B was added to the wells for 1 h each at 37 °C. TMB substrate reaction was proceeded for 15 min and the optical density was determined at 450 nm. Finally, the level of NfL in CSF and plasma was detected using Simoa NF-light Advantage Kit. Plasma and CSF were diluted 4-folds and 100-folds, respectively. CSF and plasma NfL were measured in the DNA Link Laboratory, South Korea, on the Simoa-HD1 platform as previously described^57^.

### Telomere length (TL) assay

DNA was extracted from the whole blood using D-DEX IIb RBC Lysis Buffer and D-DEX IIb Cell Lysis Buffer (Intron, MA, USA). DNA hydration was performed with 300 μL of DNA hydration solution (QIAGEN, Hilden, Germany). TL analysis was carried out using a nonradioactive TeloTAGGG TL Assay (Roche Boehringer-Mannheim, Grenzach-Wyhlen, Germany) according to the manufacturer’s instructions. Approximately 2–4 μg of DNA from each sample was digested with Hinf I/RsaI enzyme mix and isolated by gel electrophoresis. DNA fragments were transferred to a nylon membrane (Millipore, Bedford, MA, USA) by Southern transfer and hybridized to digoxigenin (DIG)-labeled probes specific for telomeric repeats. The membrane was incubated with DIG-specific antibodies conjugated to alkaline phosphatase, and the probe was visualized by chemiluminescence detection and an image analyzer (ImageQuant LAS 4000; GE Healthcare, Little Chalfont, UK). The mean telomeric repeat binding factor lengths were determined by comparing them to the molecular weight standards.

### Statistical analysis

The statistical analyses included a χ^2^ test to compare group differences in dichotomous variables, such as sex, diabetes, hypertension, and dyslipidemia, between two groups including the effect of the *APOE* genotype. To compare the levels of CSF Aβ_42_, T-tau, and P-tau S199 between amyloid positive and negative groups, Mann–Whitney test was used. Linear regression analyses were used to demonstrate a correlation between the CSF and plasma biomarkers. We defined a p-value less than 0.05 was statistically significant (SPSS Version 21.0; IBM Corp., Armonk, NY, USA).
